# Quantitative assessment of collateral time on perfusion computed tomography in acute ischemic stroke patients

**DOI:** 10.3389/fneur.2023.1230697

**Published:** 2023-08-24

**Authors:** Yao Xu, Jianhong Yang, Xiang Gao, Jie Sun, Qing Shang, Qing Han, Yuefei Wu, Jichuan Li, Tianqi Xu, Yi Huang, Yuning Pan, Mark W. Parson, Longting Lin

**Affiliations:** ^1^Department of Neurology, The First Affiliated Hospital of Ningbo University, Ningbo, Zhejiang, China; ^2^Department of Neurosurgery, The First Affiliated Hospital of Ningbo University, Ningbo, Zhejiang, China; ^3^Key Laboratory of Precision Medicine for Atherosclerotic Diseases of Zhejiang Province, Ningbo, China; ^4^Department of Radiology, The First Affiliated Hospital of Ningbo University, Ningbo, Zhejiang, China; ^5^Sydney Brain Center, University of New South Wales, Sydney, NSW, Australia; ^6^Department of Neurology, Liverpool Hospital, Sydney, NSW, Australia

**Keywords:** collateral velocity, quantitative assessment, computed tomography, perfusion imaging, stroke

## Abstract

**Background and aim:**

Good collateral circulation is recognized to maintain perfusion and contribute to favorable clinical outcomes in acute ischemic stroke. This study aimed to derive and validate an optimal collateral time measurement on perfusion computed tomography imaging for patients with acute ischemic stroke.

**Methods:**

This study included 106 acute ischemic stroke patients with complete large vessel occlusions. In deriving cohort of 23 patients, the parasagittal region of the ischemic hemisphere was divided into six pial arterial zones according to pial branches of the middle cerebral artery. Within the 85 arterial zones with collateral vessels, the receiver operating characteristic analysis was performed to derive the optimal collateral time threshold for fast collateral flow on perfusion computed tomography. The reference for fast collateral flow was the peak contrast delay on the collateral vessels within each ischemic arterial zone compared to its contralateral normal arterial zone on dynamic computed tomography angiography. The optimal perfusion collateral time threshold was then tested in predicting poor clinical outcomes (modified Rankin score of 5–6) and final infarct volume in the validation cohort of 83 patients.

**Results:**

For the derivation cohort of 85 arterial zones, the optimal collateral time threshold for fast collateral flow on perfusion computed tomography was a delay time of 4.04 s [area under the curve = 0.78 (0.67, 0.89), sensitivity = 73%, and specificity = 77%]. Therefore, the delay time of 4 s was used to define the perfusion collateral time. In the validation cohort, the perfusion collateral time showed a slightly higher predicting power than dynamic computed tomography angiography collateral time in poor clinical outcomes (area under the curve = 0.72 vs. 0.67; *P* < 0.001). Compared to dynamic computed tomography angiography collateral time, the perfusion collateral time also had better performance in predicting final infarct volume (R-squared values = 0.55 vs. 0.23; *P* < 0.001).

**Conclusion:**

Our results indicate that perfusion computed tomography can accurately quantify the collateral time after acute ischemic stroke.

## Introduction

For acute ischemic stroke (AIS) patients, collateral flow helps to maintain the blood supply to the brain region when the main artery supplying the region is occluded (1). Collateral flow after cerebral occlusion plays an important role in preventing ischemic tissue from progressing to infarction and can be a marker of the ischemic penumbra (salvageable brain tissue) (2). Good collateral has been reported to be an independent predictor of small infarct volumes (3, 4) and favorable clinical outcomes in ischemic stroke patients (5, 6). However, in the previous studies (3, 4, 7, 8), good collateral often refers to the extent of collateral flow or collateral vessels to the ischemic region.

In clinical practice, the American Society of Interventional and Therapeutic Neuroradiology/Society of Interventional Radiology (ASITN/SIR) grading system has become a commonly used approach to grade collaterals (9). It not only grades the extent of collateral flow into good and poor but also grades the speed of collateral flow into fast and slow. However, the definition of fast vs. slow collateral time is not clear. Moreover, limited evidence is available regarding the necessity of measuring collateral time. Two recent studies investigated quantitative measurements of collateral time (10, 11). Beyer's study reported that fast collateral flow was an independent predictor of small follow-up infarct lesions and infarct growth (10). In the other study, Cao's study group concluded that slow collateral flow was associated with poor radiologic outcomes and poor functional outcomes (11). However, those two studies quantified collateral time on dynamic computed tomography angiography (dCTA) (10, 11), the approach of which is user-dependent and time-consuming. A simple and valid approach is needed to quantify collateral time.

A previous study by our group showed that perfusion computed tomography (CTP) has the capacity to quantify the extent of collateral automatically (12). In this study, we planned to further explore the implementation of CTP in quantifying collateral time. The aim of this study was to derive and validate a simple and user-independent approach on CTP to quantify collateral time in acute ischemic stroke patients.

## Materials and methods

### Study design

The study consisted of two parts. The first part was to derive the optimal collateral time measurements on CTP. The second part was to compare the CTP collateral time measurement and dCTA collateral time in predicting ischemic tissue volume, imaging outcomes, and clinical outcomes. Two cohorts were used: Cohort 1 for part 1 analysis and Cohort 2 for part 2 analysis.

### Patients

**Figure 1:**
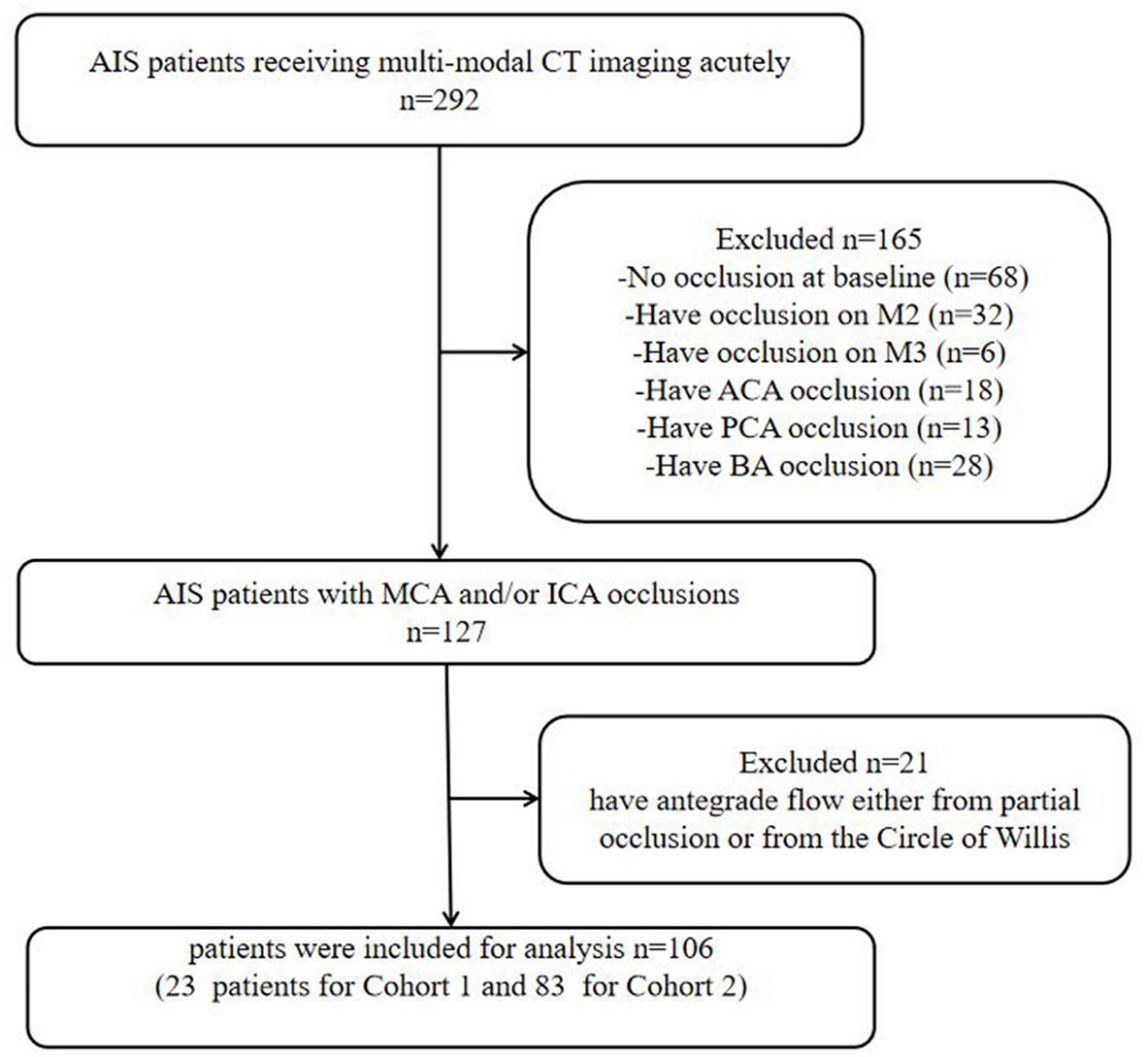
Flowchart of the inclusion of patients in the study. AIS, acute ischemic stroke; ICA, internal carotid artery; MCA, middle cerebral artery; ACA, anterior cerebral artery; PCA, posterior cerebral artery; BA, basilar artery.

This retrospective study included AIS patients between January 2017 and December 2019. Patients who presented to our center were included in this study. The study had institutional ethics approval, and written informed consent was obtained for each patient for their routinely collected data to be used in this study as part of the International Stroke Perfusion Imaging Registry (INSPIRE). All AIS patients received multi-modal CT imaging acutely. The following inclusion criteria were further applied to select patients: Baseline dCTA showed complete occlusion on the cranial segment of the internal carotid artery (ICA) or the M1 segment of the middle cerebral artery (MCA). Patients without baseline occlusion were excluded. Patients with occlusion of the M2 segment of MCA, the M3 segment of MCA, anterior cerebral artery (ACA), posterior cerebral artery (PCA), or basilar artery (BA) were also excluded. A detailed flowchart of the patient selection process is provided in Figure 1.

To ensure that CTP collateral time measurement was truly assessing retrograde flow *via* leptomeningeal collaterals, patients with antegrade flow either from partial occlusion or from the circle of Willis (*via* detection on dCTA) were excluded. To ensure the measurement of imaging and clinical outcomes, the following exclusion criteria were further applied: no follow-up imaging, hemorrhagic transformation on follow-up imaging, and no 3-month clinical outcomes recorded.

### Computed tomographic acquisition and post-processing

CT data were acquired on a 320-detector Toshiba scanner (Toshiba Aquilion ONE; Toshiba Medical Imaging, Tokyo, Japan). The multi-modal CT protocol included non-contrast computed tomography (NCCT), dCTA, and CTP. The dCTA and CTP were generated from one acquisition. Details of the acquisition process were as follows: Temporally, the acquisition started 7 s after non-ionic iodinated contrast injection into an antecubital vein (40 ml, 6 ml/s; Bayer HealthCare, Berlin, Germany) and lasted for 65 s. It consisted of three phases, resulting in 19 time frames. In phase one, 1 frame was generated (80 kV, 310 mA) as a baseline; in phase two, 13 frames were generated at a rate of one frame per 2 s (80 kV, 150/300 mA); in phase three, 5 frames were generated at a rate of one frame per 5 s (80 kV, 150 mA). Spatially, one gantry rotation resulted in 320 axial slices with a thickness of 0.5 mm, which covered 160 mm on the z-axis.

CTP data were processed by commercial software MIStar (Apollo Medical Imaging Technology, Melbourne, VIC, Australia) with a fully automated processing algorithm, applying singular value decomposition (SVD) with delay and dispersion correction (13), generating delay time (DT), delay-corrected cerebral blood flow (dCBF), delay-corrected cerebral blood volume (dCBV), and delay-corrected mean transit time (dMTT).

### CTP threshold setting

On CTP maps, ischemic lesions were delineated by setting thresholds. For DT maps, the range of threshold setting was from 0 to 10 s with increments of 0.5 s; for dCBF, the range was from 100 to 0% with 5% decrements. The threshold level was relative to the mean perfusion value of the unaffected brain tissue of the same patient. The dual threshold setting was used to define the ischemic lesion and core on CTP, with the upper threshold delineating the entire ischemic lesion and with the lower threshold defining the ischemic core. Penumbra was measured by ischemic lesion minus core volume, and mismatch ratio was defined by ischemic lesion divided by core volume. For maps generated by dSVD, ischemic lesion and core were defined by DT >3 s and dCBF < 30% (14), respectively.

#### Part 1: arterial zone analysis: deriving the optimal collateral time measure on CTP

**Figure 2:**
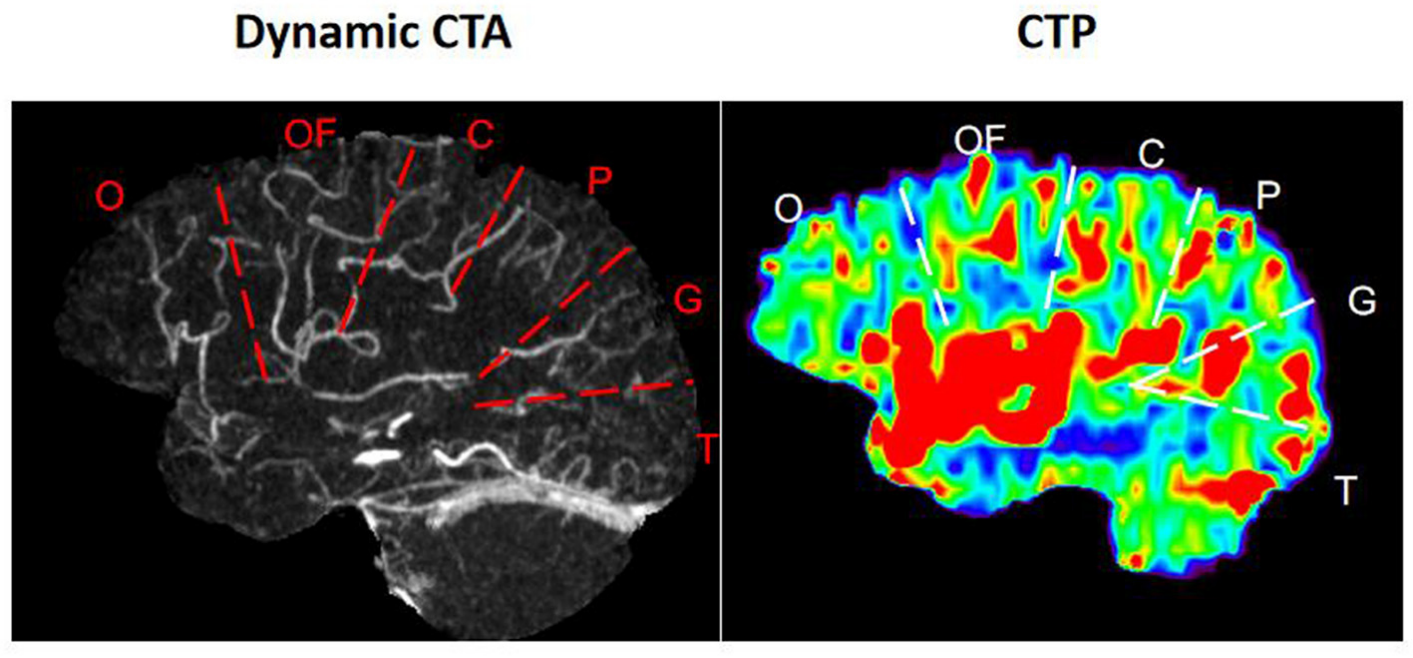
Arterial zones of the middle cerebral artery. O, orbitofrontal arteries; OF, operculofrontal arteries; C, central arteries; P, anterior and posterior parietal arteries; G, gyrus angularis arteries; T, temporal arteries.

**Figure 3:**
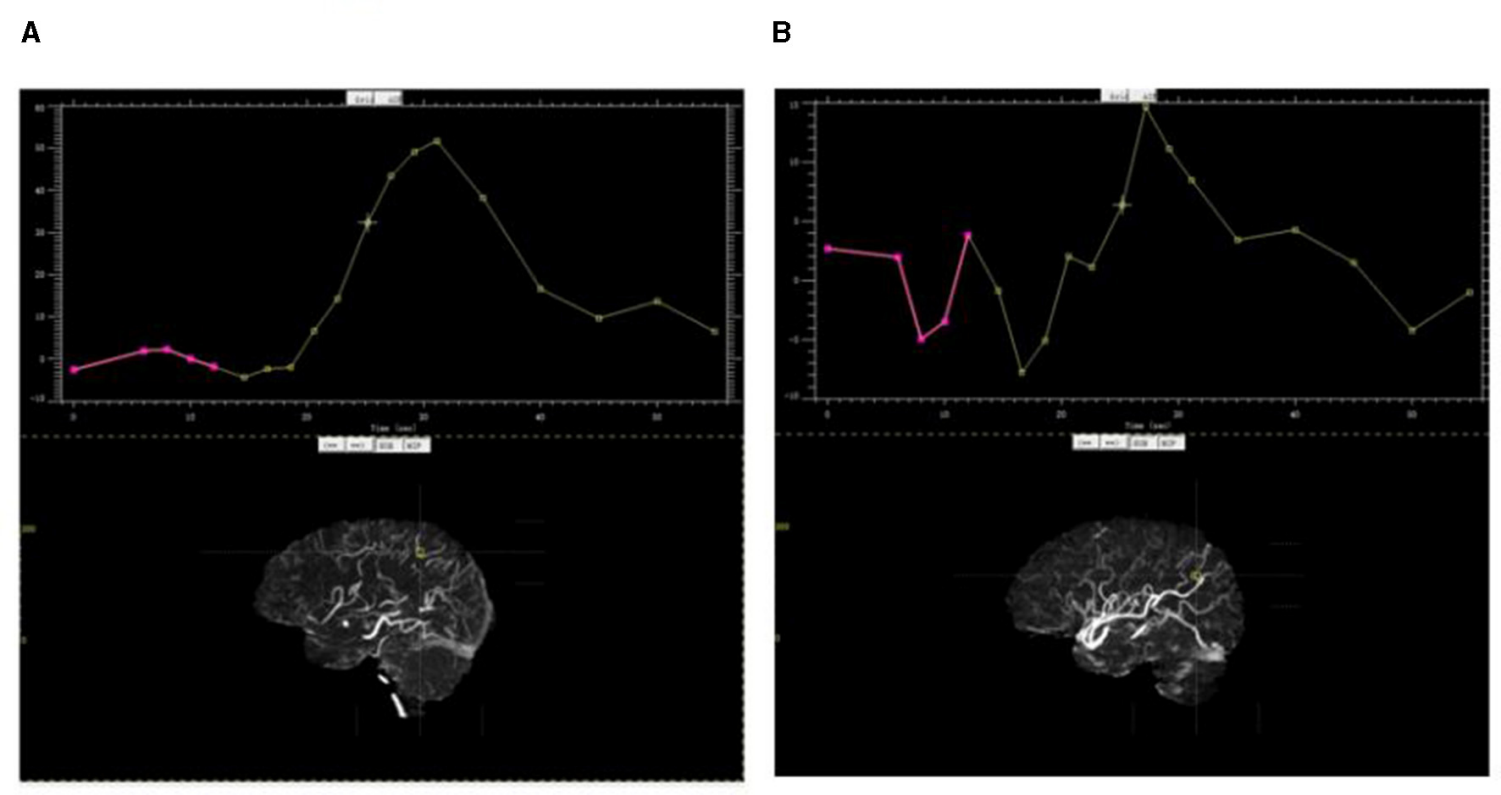
Contrast peak time by the time vs. contrast density curve. **(A)** Contrast peak time in the parietal arterial zone of the ischemic hemisphere; **(B)** contrast peak time in the same site in the contralateral arterial zone of the unaffected hemisphere.

**Figure 4:**
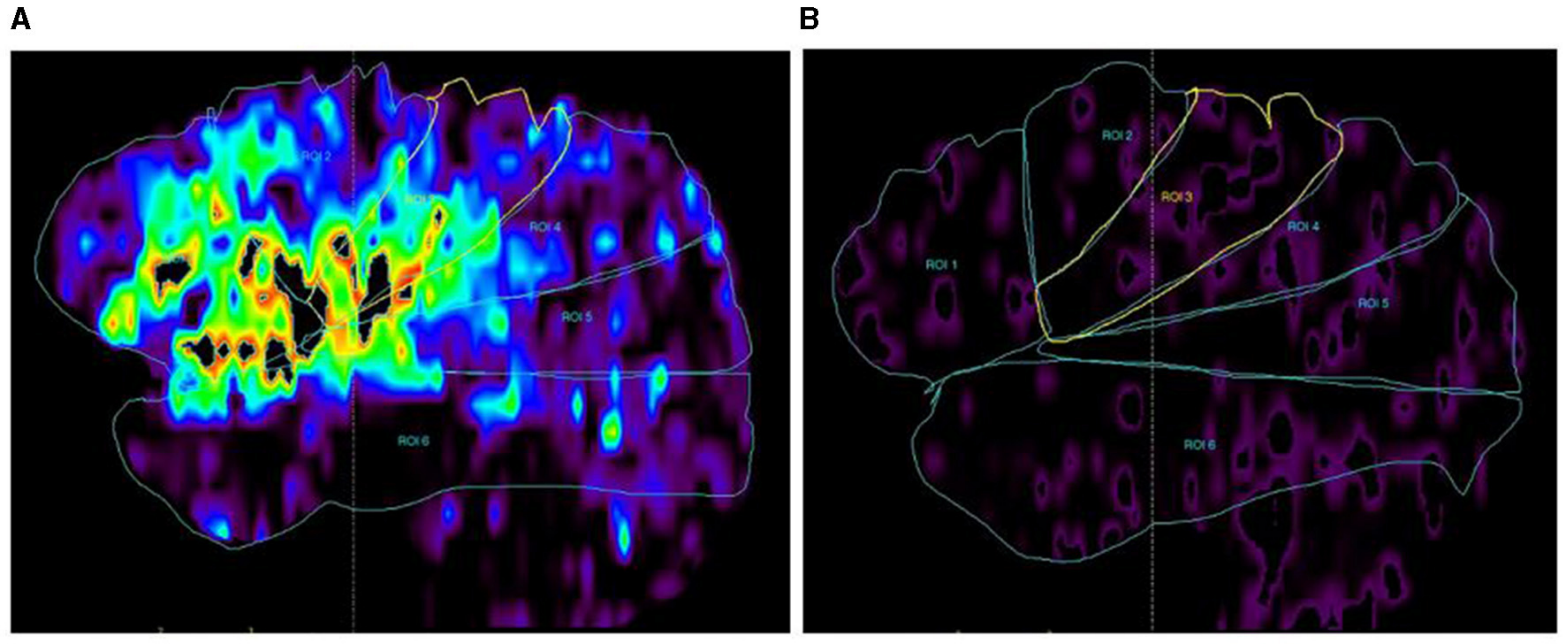
CTP map (DT map) by ROC analysis. **(A)** DT map in each arterial zone of the ischemic hemisphere; **(B)** DT map in each arterial zone of the unaffected hemisphere. DT values in each arterial zone were measured on the DT map automatically, and so did the CBF, CBV, and MTT.

1) On dCTA and CTP of Cohort 1, for each hemisphere, the parasagittal region was divided into the following six zones according to the middle cerebral artery (MCA) distal cortical branches (Figure 2): orbitofrontal arteries (O), operculofrontal arteries (OF), central arteries (C), anterior and posterior parietal arteries (P), gyrus angularis arteries (G), and temporal arteries (T). For each ischemic arterial zone, the presence or absence of pial vessel filling was recorded on dCTA. For the hemisphere affected by ischemia, regions with retrograde filling of pial arteries were considered as having flow from the leptomeningeal collaterals. The approach was adopted from methods previously described to classify pial branches of MCA and corresponding arterial territories (15). It is based on the principle that the same pial pathway would be used by collateral flow when the MCA is occluded. Therefore, the ischemic arterial zones were further divided into collateral arterial zones (with pial vessels from retrograde collateral flow in ischemic hemispheres) and non-collateral arterial zones (without pial vessels in ischemic hemispheres).
The non-collateral arterial zones were excluded from this study. For each collateral arterial zone, the collateral time was measured on dCTA (the contrast delayed peak time compared to its contralateral normal arterial zone) and classified into the following four groups: < 2 s, 2–4 s, 4–6 s, and >6 s. It was further dichotomized into fast and slow collateral flow. The arterial zones with fast collateral flow appeared to have a limited ischemic core.
2) dCTA collateral time measurement
For each collateral arterial zone of the ischemic hemisphere, the region of interest was manually placed at the most prominent collateral vessel near the Sylvian fissure, and the contrast peak time could be taken by the time vs. contrast density curve. The contrast peak time was also taken in the same site in the contralateral arterial zone of the unaffected hemisphere. The dCTA collateral time was defined as the contrast delayed peak time of the ischemic arterial zone compared to its contralateral normal arterial zone (Figure 3).
3) For each collateral arterial zone, perfusion values were measured on CTP automatically by region of interest analysis (ROI analysis). Six regions were drawn of the ischemic hemisphere according to the MCA distal cortical branches also (Figure 4). Therefore, we got every CTP perfusion region corresponding to each collateral arterial zone, and all values of CTP parameters in each collateral arterial zone could be obtained automatically using MIStar software. Then, the optimal CTP perfusion threshold for differentiating fast from slow collateral flow was further obtained.

A neurologist with approximately 10 years of experience (Y.X.) and another neurologist with approximately 8 years of experience (Q.S.), blinded to clinical and imaging outcomes, independently and retrospectively evaluated each arterial zone. The divisions of arterial zones and measurements of dCTA collateral time were compared and regarded by consensus where independent grading differed. The proportion of agreement between readers was calculated using κ statistics.

#### Part 2: patient analysis: comparing collateral time measured by CTP and dCTA

The optimal CTP perfusion threshold from Cohort 1 was then used to define CTP collateral time. The CTP collateral time and dCTA collateral time were compared in predicting the final infarct volume and 3-month clinical outcome. The final infarct volume was measured on 24–72 h NCCT or magnetic resonance diffusion-weighted imaging.

### Clinical outcome

Modified Rankin Score (mRS) at 3 months was the primary clinical outcome of patients. Patients were divided into those with poor clinical outcomes and those without poor clinical outcomes (mRS of 5–6 vs. 0–4).

### Statistical analysis

Patient characteristics were summarized by the median and interquartile range (IQR) if they were continuous data and by proportion if they were categorical or binary data. For continuous variables, the Mann–Whitney U-test was used to compare differences between the two groups. For categorical variables, the χ^2^ test was used to compare differences among groups. All statistical analyses were performed using STATA 13.0 (Stata Corp, College Station, Texas, USA), with a confidence interval (CI) set at 95% and a significant level set at 0.01.

For part 1 analysis, the median core volume was plotted across the dCTA collateral time. The fast collateral flow was defined by the dCTA collateral time that predicted no ischemic core lesion within the arterial zone. Then, the correlation of CTP parameter values and dCTA collateral time was evaluated using Spearman's rank correlation analysis, and the optimal CTP perfusion threshold for differentiating fast from slow collateral flow was obtained using the receiver operating characteristic (ROC) analysis and the Youden Index.

For part 2 analysis, simple regression models were used to assess the predictive power of the CTP collateral time and dCTA collateral time in final infarct volume. The predictive power was assessed by R-square value and *p*-value. To assess the performance of the CTP collateral time and dCTA collateral time in predicting clinical outcomes, simple logistic regression models were performed, followed by ROC analysis. The predictive power was assessed by the area under the curve (AUC) and *p*-value.

Additionally, the following sensitivity analyses were performed: (1) Repeating the analysis of part 1 in a new group of patients from another INSPIRE center. (2) Processing CTP with the standard deconvolution approach. The following CTP parameters were generated with standard SVD without delay and dispersion correction: standard CBF (sCBF), standard CBV (sCBV), standard MTT (sMTT), and time-to-maximum of the residual function (Tmax).

## Results

### Patients

A total of 292 AIS patients were recruited from our center. After applying the patient selection criteria (detailed in Figure 1), 106 were selected. Of these, 23 were selected for Part 1 analysis (Cohort 1), and the remaining 83 patients were used for Part 2 analysis (Cohort 2). Patient characteristics in Cohort 1 and Cohort 2 are summarized in Table 1.

**Table 1:** Patient characteristics.

**Patient characteristics**	**Cohort 1 (*n =* 23)**	**Cohort 2 (*n =* 83)**	***P***
Age, median (IQR)	73 (61–84)	75 (66–82)	0.702
Male, % (*N*)	60.9 (14)	59.0 (49)	0.762
Baseline NIHSS, median (IQR)	17 (15–19)	15 (10–19)	0.207
Baseline perfusion volume (ml), median (IQR)	158.1 (105.9–205.6)	116.1 (71.1–157.4)	0.026
Baseline core volume (ml), median (IQR)	65.7 (13.5–115.4)	23.1 (9.1–57.9)	0.034
Baseline penumbra volume (ml), median (IQR)	86.1 (55.4–113.4)	81.0 (53.4–110)	0.540
Baseline mismatch ratio, median (IQR)	2.7 (1.7, 5.9)	4.3 (2.6–9.6)	0.116
Onset to image time (hours), median (IQR)	2.8 (2–3.5)	2.5 (2–3.8)	0.647
Intravenous thrombolysis rate, % (*N*)	69.6 (16/23)	63.9 (53/83)	0.084
Onset to needle time (hours), median (IQR)	3.2 (2.4–4)	2.8 (2.2–3.7)	0.143
Endovascular thrombectomy, % (*N*)	0 (0/23)	37.3 (31/83)	< 0.001
Onset to groin time (hours), median (IQR)	NA	3.8 (2.5–7.3)	NA
Onset to recanalization time (hours), median (IQR)	NA	5.3 (4.1–9.4)	NA
Recanalization, % (*N*)	39.1 (9/23)	65.1 (54/83)	0.031
24-h NIHSS, median (IQR)	16 (12–19)	8 (4–15)	0.006
Final infarct volume (ml), median (IQR)	106.6 (15.9–161.6)	39.1 (10.1–120)	0.005
Poor outcome rate, % (*N*)	60.9 (14/23)	32.5 (27/83)	0.074

IQR refers to interquartile range; NIHSS refers to National Institute Health Stroke Scale; baseline penumbra and core volume are measured on CTP; final infarct volume is measured on follow-up diffusion-weighted imaging or non-contrast CT at 24 h; recanalization is defined by a Thrombolysis in Cerebral Infarction (TICI) score of 2b-3 after endovascular procedure for endovascular thrombectomy (EVT) patients and on follow-up CTA or magnetic resonance imaging for non-EVT patients; poor outcome is defined by modified Rankin score of 5–6 at 3 months.

#### Part 1: deriving the optimal collateral time measurement on CTP

##### Classifying contrast delayed peak time on dCTA

**Figure 5:**
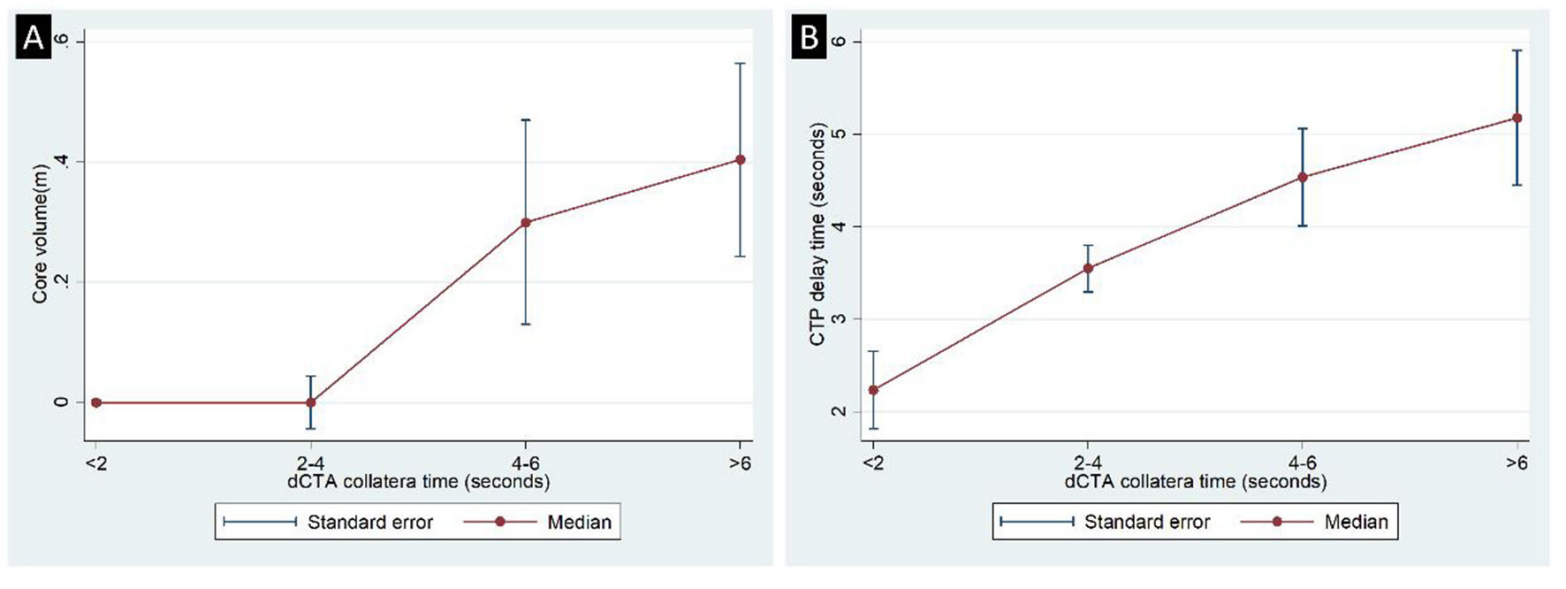
Distribution of CTP core volume **(A)** and delay time **(B)** across dCTA collateral time.

Part 1 analysis included 23 patients, resulting in 138 ischemic arterial zones. Of the 138 ischemic arterial zones, 85 had collateral vessels and 53 did not have collateral vessels. For the 85 arterial zones with collateral vessels, with the increase of dCTA collateral time, acute core volume increased significantly (*P* < 0.001, Figure 5A). For patients with dCTA collateral time of < 2 s or 2–4 s, the median core volume was 0 mL. The ROC analysis showed that the contrast delayed peak time of 4 s was the optimal cut point for predicting a core volume of 0 ml [AUC = 0.74 (0.61, 0.81)]. Based on this finding, we classified the collateral time into fast (< 4 s contrast delayed peak time) and slow (≥4 s contrast delayed peak time) on dCTA.

##### Deriving the optimal CTP threshold for fast collateral flow

For the 85 ischemic arterial zones, the CTP parameter of DT showed a significant correlation with dCTA collateral time (correlation coefficient = 0.51, p < 0.001, Figure 5B). The median DT time was 2.23 s (1.07–2.65) for the dCTA collateral time < 2 s, 3.54 s (2.22–4.41) for the dCTA collateral time of 2–4 s, 4.54 s (3.81–5.73) for the dCTA collateral time of 4–6 s, and 5.18 s (4.37–6.19) for the dCTA collateral time >6 s. dMTT and dCBF also had significant correlations with dCTA collateral time (correlation coefficient = 0.41 and −0.35 respectively; *P* < 0.001).

**Figure 6:**
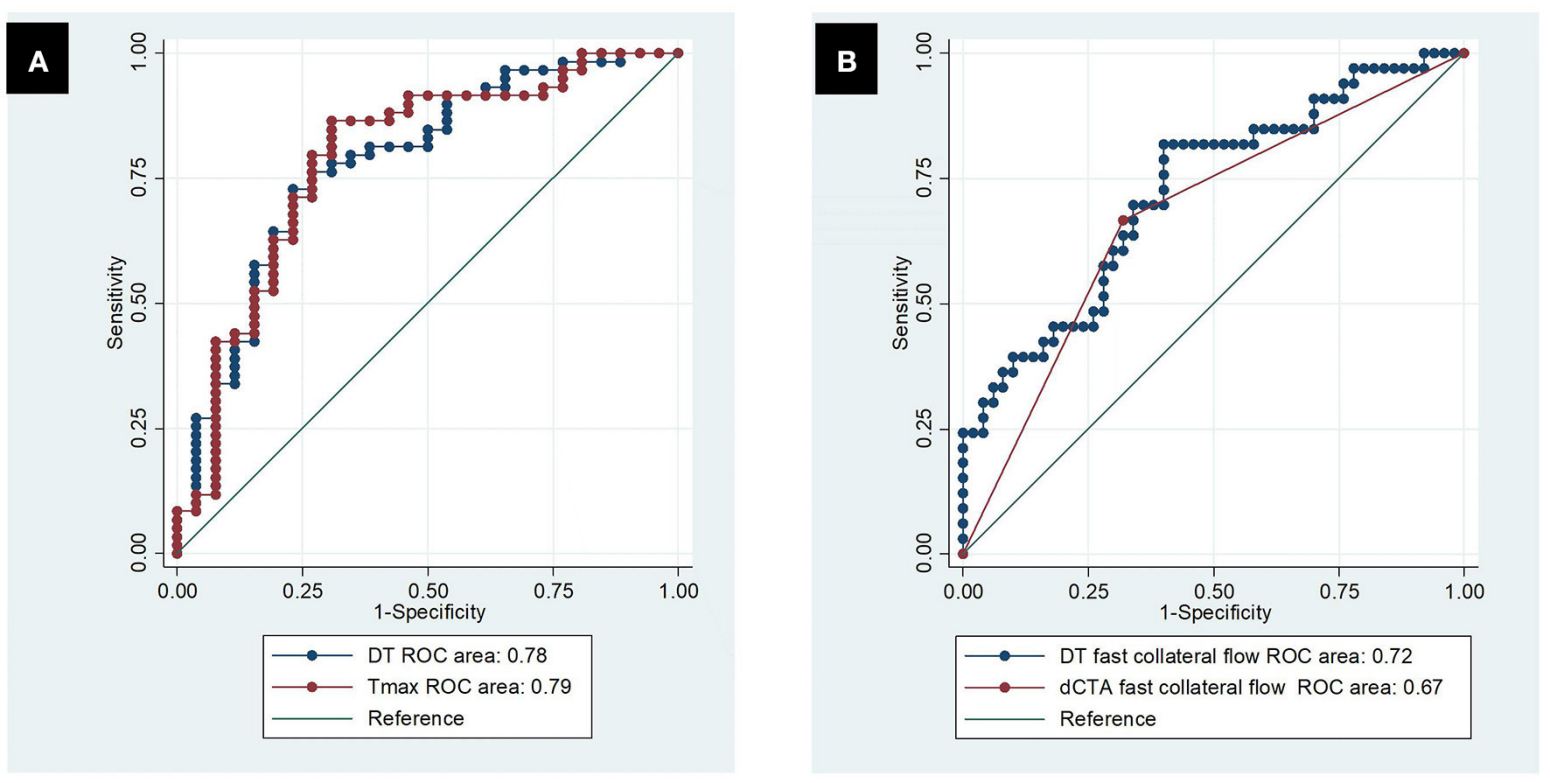
Receiver operating characteristic curve. **(A)** Predicting dCTA fast collateral flow by DT and Tmax; **(B)** predicting poor clinical outcomes by dCTA slow collateral flow and DT slow collateral flow.

DT, compared to dMTT/dCBF/dCBV, had the largest ROC curve area in differentiating between the arterial zones of fast collateral flow and slow collateral flow [AUC = 0.78 (0.67–0.89), Table 2, Figure 6A]. DT at the threshold of 4.04 s had the maximum sensitivity and specificity (73 and 77%, respectively) in differentiating between fast and slow collateral arterial zones.

**Table 2:** Optimal CTP parameters in predicting fast collateral flow in Cohort 1.

**CTP parameters from dSVD**	**AUC**	**Optimal threshold**	**Sensitivity**	**Specificity**
DT (seconds)	0.78 (0.67, 0.89)	4.04	73%	77%
dMTT (seconds)	0.76 (0.66, 0.87)	8.81	71%	77%
dCBF (ml/min/100 g)	0.70 (0.56, 0.84)	16.97	86%	58%
dCBV (ml/100 g)	0.56 (0.42, 0.70)	2.73	61%	65%
**CTP parameters from sSVD**	**AUC**	**Optimal threshold**	**Sensitivity**	**Specificity**
Tmax (seconds)	0.79 (0.68, 0.90)	7.26	86%	69%
sMTT (seconds)	073 (0.62, 0.85)	11.94	76%	65%
sCBF (ml/min/100 g)	0.71 (0.58, 0.84)	12.81	88%	58%
sCBV (ml/100 g)	0.57 (0.43, 0.71)	2.73	64%	65%

AUC, area under the curve; CTP, computed tomographic perfusion; CBF, cerebral blood flow; CBV, cerebral blood volume; MTT, mean transit time; and DT, delay time.

#### Part 2: CTP collateral time predicting patient outcomes

In part 2, based on analysis in part 1, DT of 4 s was established to differentiate fast from slow collateral flow on CTP. For the 83 patients of cohort 2, DT collateral time and dCTA collateral time had a good correlation (correlation coefficient = 0.45, *P* < 0.001) on the patient level.

The simple logistic regression models showed that collateral velocity measured either by DT collateral time or dCTA collateral time strongly predicted poor clinical outcomes (*P* < 0.001). The DT collateral time showed slightly higher predicting power than the dCTA collateral time in poor clinical outcomes (AUC = 0.72 vs. 0.67, Figure 6B). The simple regression models showed that either the DT collateral time or dCTA collateral time predicted final infarct volume significantly (*P* < 0.001). DT collateral time also had better performance than dCTA collateral time in predicting final infarct volume (R-square of 0.55 vs. 0.23).

### Sensitivity analysis

When repeating the analysis of part 1 in a new cohort (Cohort 3), DT among the four CTP parameters had the largest area under the ROC curve [0.91 (0.86–0.96)]. The optimal cutpoint of DT is 3.26 s (sensitivity = 82%, specificity = 91%) (Supplementary Figure S1). The new cohort included 19 patients, of whom 14 (73.7%) had endovascular thrombectomy, whereas no endovascular thrombectomy was performed in the derivation cohort in this study (Cohort 1). The new cohort also had patients presenting later than the deriving cohort (median onset to imaging time of 5 h vs. 2.8 h) (Supplementary Table S1).

For CTP parameters processed by standard SVD, Tmax had similar performance to DT in terms of predicting fast vs. slow collateral flow on dCTA [AUC = 0.79 (0.68, 0.90) vs. 0.78 (0.67–0.89), Figure 6A, Table 2]. The optimal threshold of Tmax was 7.26 s with a sensitivity of 86% and specificity of 69% in defining fast vs. slow collateral flow.

## Discussion

This study was the first to derive and validate a quantitative assessment for collateral time on CTP. We found that a delay time of 4 s had the highest power in predicting fast collateral flow. According to this study, the DT collateral time (DT > 4 s) can well predict lesion outcomes and poor clinical outcomes after ischemic stroke. This DT collateral time can be fully automated and obtained by computer processing. Therefore, this study provides an automated, user-independent, and quantitative assessment of collateral time.

This study confirms the importance of collateral time and provides evidence to support 4 s as the optimal threshold to distinguish fast from slow collateral flow on dCTA. The ASITN/SIR grading score is widely used for collateral assessment on dCTA; it evaluates both the time and extent of the collaterals. Compared with other CTA collateral scores, the ASITN/SIR has been reported to have better predictive power for the image and clinical outcomes (3, 16). The challenge to grade collaterals with ASITN/SIR is how to define fast vs. slow collateral flow. One study suggested that contrast peak time delay in the affected territory > 4 s might reflect slow collateral flow on dCTA since this threshold had a correlation with poor clinical outcomes (11). Moreover, some studies based on CTP and collaterals have been reported recently. One study showed that hypoperfusion index (defined by Tmax > 10 s/Tmax > 6 s volume) >0.5 was able to distinguish fast from slow in the initial rate of core progression in acute ischemic stroke patients. How the optimal CTP parameter and its threshold were established is not described (17). In this study, we used tissue outcomes to validate the contrast peak time delay of 4 s as the optimal threshold differentiating fast and slow collaterals on dCTA. In this study, for patients with fast collateral of < 4 s, the majority of the ischemic lesion would be penumbra and it resulted in limited ischemic core volume.

An important clinical application of this study is providing an automated and quantitative measure of collateral time. CTP showed similar or even better performance than CTA in assessing the collateral time after acute ischemic stroke in this study. In clinical application, compared to dCTA, CTP collateral time has the following advantages. First, CTP collateral measurement is user-independent, whereas collateral assessment on dCTA depends on the experience of the observer. At present, the ASITN/SIR grading system is the most commonly used approach to assess collaterals on dCTA. It is a semi-quantitative classification with poor inter-observer agreement, even among experienced observers. According to a previous study about the ASITN/SIR grading system, collateral grading varied widely between observers regardless of their experience: slight for >10 years' experience (κ = 0.13) and fair for < 5 years' experience (κ = 0.28); grade 4 showed the worst degree of inter-observer agreement (κ = 0.18), whereas grade 0 or 1 were associated with the best agreements (κ = 0.52; κ = 0.43, respectively) (18). This significant inter-observer variation might lead to bias in selecting the “right” patient to treat (5, 6). The inter-observer variation of collateral time assessment can be addressed by the user-independent approach to CTP. Second, CTP collateral time quantification can be generated automatically and in a timely manner. The automated processing of CTP is fast (< 10 min). This helps to address what might be time-consuming manual scoring of collaterals (19). Third, CTP is a quantitative way of measuring collateral time.

This is not the first study to assess collateral time. However, there are two important novelties of this study. First, this study systematically analyzed the dCTA collateral time (delayed peak time compared to the contralateral) and all perfusion parameters to derive the optimal perfusion parameter and thresholds to quantify collateral time. In comparison, no previous study could obtain or validate a threshold to distinguish fast collaterals from slow collaterals on dCTA (10, 11, 20). Second, this study used a novel anatomic approach to establish the relationship between perfusion parameters and collateral flow; that is, regional comparison based on individual collateral vessels. The approach is based on methods previously described to classify pial branches of MCA and corresponding arterial zones (15). In this study, the same classification was adopted to define collateral vessels and zones since the same pial pathway would be used by retrograde collateral flow when there is no antegrade blood flow on MCA in ischemic stroke. The regional analysis in this study has the advantage of assessing individual collateral vessels, which provides more information on collateral time in comparison to previous studies assessing the collateral status of the whole brain (21). This additional collateral information could potentially explain the very high accuracy of the CTP collateral time thresholds in this study.

Limitations of this study include the following: First, this study is limited to acute ischemic stroke patients with MCA or the cranial segment of ICA occlusion without antegrade flow from the circle of Willis; for patients with ACA, PCA, or basilar occlusion, different collateral pathways are involved (22); thus, patients might require a different threshold for collateral time on CTP. Second, patients in cohort 1 were mostly imaged within 4.5 h from stroke onset and mainly received intravenous thrombolysis treatment. However, endovascular treatment is routine care for acute large vessel occlusion patients now, especially for those outside the time window (23), but it was not performed routinely during the recruitment period for this study. Studies have shown that collateral flow predicted response to both intravenous thrombolysis (5, 24) and endovascular treatment for acute ischemic stroke (6, 24). There is no reason to suspect that the CTP collateral time derived from this study would also be useful in patients' selection for endovascular thrombectomy, but obviously, further studies are required. Therefore, in the sensitivity analyses, we tested in new group patients including receiving endovascular thrombectomy treatment (Supplementary material). This can help to generalize the findings of this study. Finally, there were some imbalances between Cohort 1 and Cohort 2 in endovascular thrombectomy treatment, 24-h NIHSS, and final infarct volume. Since this article is a further study based on our previous study, we used the same Cohort 1 and made further analysis. The earlier study contained fewer patients treated with thrombectomy, which led to some differences between Cohort 1 and Cohort 2. Moreover, we just analyzed the preliminary predictive ability of the DT collateral time in this study. Larger and multicenter cohorts and further multivariate regression analysis are needed to confirm this assessment and its application in clinical practice, and we will continue to conduct in-depth research.

In conclusion, CTP provides a user-independent, quantitative, and reliable measurement of collateral time after acute ischemic stroke.

## Data availability statement

The raw data supporting the conclusions of this article will be made available by the authors, without undue reservation.

## Ethics statement

The studies involving human participants were reviewed and approved by John Hunter Hospital and Ningbo First Hospital. The patients/participants provided their written informed consent to participate in this study.

## Author contributions

Study design, organization, execution, statistical analysis, and writing of the manuscript draft: YX. Research project conception, study design, and project administration: JY. Patients' enrolment and follow up and acquisition of data: QS, QH, YW, JL, and TX. Image technology guidance: YP. Funding acquisition: XG, JS, and YH. Research project conception, study design, statistical analysis, review and critique, and manuscript revision of the draft: LL and MP. All authors contributed to the article and approved the submitted version.
