# Supplementary material for: Quantitative assessment of collateral time on perfusion computed tomography in acute ischemic stroke patients
Source: Front Neurol. 2023 Aug 24;14:1230697. doi: 10.3389/fneur.2023.1230697 (PMC10491895; doi:10.3389/fneur.2023.1230697)
Supplement: Supplementary file 1 [file Data_Sheet_1.pdf]

SUPPLEMENTARY MATERIAL

Quantitative Assessment of Collateral Time on Perfusion Compute Tomography in Acute Ischemic Stroke Patients

Yao Xu[1,†], Dr; Jianhong Yang[1,†], Dr; Xiang Gao[2], MD; Jie Sun[2], MD; Qing Shang[1], Dr; Qing Han[1], MD; Yuefei Wu[1], Dr; Jichuan Li[1], Dr; Tianqi Xu[1], Dr; Yi Huang[2, 3], PhD; Yuning Pan[4], MD; Mark W. Parson[5, 6,*], PhD, MD; Longting Lin[1,5,*], PhD

1 Department of Neurology, The First Affiliated Hospital of Ningbo University, Ningbo, Zhejiang, China;

2 Department of Neurosurgery, The First Affiliated Hospital of Ningbo University, Ningbo, Zhejiang, China;

3 Key Laboratory of Precision Medicine for Atherosclerotic Diseases of Zhejiang Province, Ningbo, China;

4 Department of Radiology, The First Affiliated Hospital of Ningbo University, Ningbo, Zhejiang, China;

5 Sydney Brain Center, University of New South Wales, Sydney, New South Wales, Australia;

6 Department of Neurology, Liverpool Hospital, Sydney, New South Wales, Australia.

# Corresponding Author:

Longting Lin, PhD, Sydney Brain Center, University of New South Wales, New South Wales, Australia. Email: longting.lin@unsw.edu.au

Mark W. Parsons, PhD, MD, Sydney Brain Center, University of New South Wales, New South Wales, Australia. Email: Mark.Parsons@unsw.edu.au

†These authors contributed equally to this work

Supplementary Table 1. Patient characteristics

Patient characteristics	Cohort 3 (n=19)
Age, Median (IQR)	70(59-79)
Male, % (N)	57.9(11)
Baseline NIHSS, Median (IQR)	19(12-21)
Baseline perfusion volume (ml), Median (IQR)	107(85.5-124.5)
Baseline core volume (ml), Median (IQR)	25.6(10.3-68)
Baseline penumbra volume (ml), Median (IQR)	82.2(61-108)
Baseline mismatch ratio, Median (IQR)	6(3.8-11.3)
Onset to image time (hours), Median (IQR)	5(3.25-9)
Intravenous thrombolysis rate, % (N)	26.3(5/19)
Onset to needle time (hours), Median (IQR)	3.1(2.5-4.1)
Endovascular thrombectomy, % (N)	73.7(14/19)
Onset to groin time (hours), Median (IQR)	6.9(4.6-10.2)
Onset to recanalization time (hours), Median (IQR)	7.9(5.2-11)
Recanalization, % (N)	68.4(13/19)
24-hour NIHSS, Median (IQR)	13(6-19)
Final infarct volume (ml), Median (IQR)	35.7(19-106)
Poor outcome rate, % (N)	21.1(4/19)

IQR refers to interquartile range; NIHSS refers to National Institute Health Stroke Scale; baseline penumbra and core volume are measured on CTP; final infarct volume is measured on follow-up diffusion-weighted imaging or non-contrast CT at 24 hours; recanalization is defined by a Thrombolysis in Cerebral Infarction (TICI) score of 2b-3 after endovascular procedure for Endovascular thrombectomy (EVT) patients and on follow-up CTA or magnetic resonance imaging for non-EVT patients; poor outcome is defined by modified Rankin score of 5-6 at 3 months.

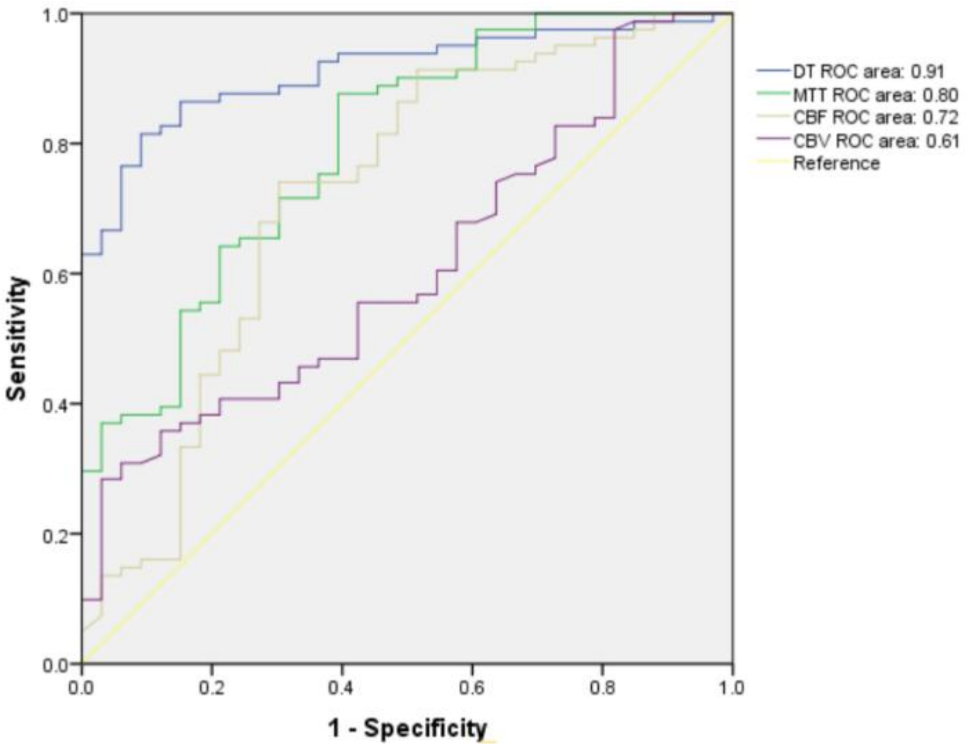

Supplementary Figure 1. Receiver operating characteristic (ROC) curves of CTP parameters in differentiating fast and slow collateral flow in arterial zones of Cohort 3. DT, among the four CTP parameters, had the largest area under ROC curve (0.91 [0.86-0.96]). The optimal cutpoint of DT is 3.26 seconds (sensitivity=82%, specificity=91%)
